# Measuring functioning among youth using the Columbia impairment scale: investigating dimensionality and measurement invariance among 14–17 year olds using mental health services and their caregivers

**DOI:** 10.1186/s12888-025-06511-1

**Published:** 2025-03-19

**Authors:** Kristin Cleverley, Sarah Brennenstuhl, Peter Szatmari, Lisa D. Hawke, Karolin R. Krause, Amy Cheung, Jacqueline Relihan, Mahalia Dixon, Jo Henderson

**Affiliations:** 1 Lawrence S. Bloomberg Faculty of Nursing and Department of Psychiatry, Faculty of Medicine, University of Toronto, Toronto, Canada; 2 Margaret and Wallace McCain Centre for Child, Youth and Family Mental Health, Centre for Addiction and Mental Health, Toronto, ON Canada; 3 Student Mental Health Initiatives, University of Toronto, Toronto, Canada; 4 Cundill Centre for Child and Youth Depression, Centre for Addiction and Mental Health, Hospital for Sick Children, Temerty Faculty of Medicine, University of Toronto, Toronto, Canada; 5 Centre for Complex Interventions, Centre for Addiction and Mental Health, Toronto, Canada; 6 Department of Psychiatry, Faculty of Medicine, University of Toronto, Toronto, Canada; 7 Cundill Centre for Child and Youth Depression, Centre for Addiction and Mental Health, Toronto, Canada; 8 Department of Psychiatry, Sunnybrook Health Sciences Centre, University of Toronto, Toronto, Canada; 9 Youth Wellness Hubs Ontario, Toronto, Canada

**Keywords:** Functioning, Youth, Mental health, Structural equation modelling, Measurement invariance

## Abstract

**Background:**

Despite being a widely used and recommended measure of functioning, the Columbia Impairment Scale (CIS) lacks consensus on scale structure and whether child- and parent-report versions measure the same construct(s). This study aimed to better understand the structure and test for measurement invariance across groups of youth and their caregivers.

**Methods:**

The sample included youth 14–17 years of age accessing mental health services, and their caregiver (most often mother), recruited from one of five mental health outpatient hospital sites in Toronto, Canada between September 2016 and March 2020. Exploratory Structural Equation Modeling (ESEM) was used to investigate dimensionality and test for measurement invariance using standard model fit statistics.

**Results:**

A total of 189 youth-caregiver dyads were included in the analysis. Youth were on average aged 15.7 (sd = 1.1); 64% were female. Caregivers had a mean age of 48.2 (sd = 7.4) and were 87% mothers. Using ESEM, evidence of a three-factor model was found (“work/school”, “home/family” and “socializing”), which included several, large conceptually relevant cross-loadings. Using this model, full metric invariance between youth and caregivers was established, but strong evidence of scalar invariance was not found.

**Conclusions:**

While a multi-dimensional model provided the best fit for the CIS, the presence of several large cross-loadings calls into question whether and how the global scale can best be used in clinical and research settings. Lack of evidence of scalar invariance suggests that multi-informant data should be interpreted carefully. Next steps should include testing for essential unidimensionality.

Measuring psychiatric symptoms and their impact on social and educational or occupational domains of life is crucial to the assessment of severity and prognosis of psychiatric illness among youth. In child and adolescent psychiatry, functioning has been defined as the “capacity to adapt to the demands of home, school, and community” [1]. Determining if symptoms cause impairment in daily functioning and across these different contexts is central to the Diagnostic and Statistical Manual Version 5 (DSM-5) clinical significance criterion [2]. The assessment of functioning for distress or impairment is critical to determining need for treatment and evaluating treatment outcomes [2–5]. This is particularly important for children and youth who may experience significant distress and impairment even without meeting the threshold for disorder based on psychiatric symptoms alone [6].

Recognizing the need for a measure of child and youth functioning that includes self-assessment and assessment by a proxy (e.g. a parent or caregiver), and captures impairment separate from psychiatric symptoms, Bird et al. [7] developed the 13-item Columbia Impairment Scale (CIS). The scale was created to assess impairment in four dimensions of functioning: interpersonal relations, certain broad areas of psychopathology, functioning at school or work, and use of leisure time. Since its development in the early 1990s, the measure has been widely used and is suggested as a common measure to be implemented in child and adolescent research funded by the National Institute of Mental Health, highlighting that it is a brief, low burden and self-report measure [8, 9]. Despite the demonstrated utility of the scale, its structural validity remains largely unexplored. Initial empirical testing of scale dimensionality led the developers to conclude that the CIS is unidimensional based on Exploratory Factor Analysis (EFA) [7, 10]. Three additional studies have attempted to further validate the scale structure [11–13]. Two of these studies [11, 12] also exclusively used EFA. Drawing on a population-based sample, Attell and colleges found evidence of a unidimensional solution based on the Kaiser rule, although they that observed up to three factors were identifiable on a Scree plot [11]. Using a sample of youth aged 16–24 accessing substance use services, our previous research demonstrated a one-factor model based on parallel analysis and a Scree plot [12]. However, model fit statistics suggested less than adequate fit for the unidimensional model (RMSEA = 0.112; SRMR = 0.09) EFA is a technique used to reveal the number of latent constructs that are needed to explain the correlations among a set of scale item scores, without prior hypotheses about scale structure [14]. While useful for providing initial information about dimensionality, EFA is limited, in part because it is considered an exploratory method for use when there are no a priori assumptions about the model of the data [15]. Confirmatory Factor Analysis (CFA), on the other hand, studies hypothesized relationships between the item scores and latent constructs [14], and can be used to test alternative models of the data. This is considered fundamental to theory building and key to establishing construct validity [16]. In the only study we are aware of using CFA to validate the structure of the CIS, Singer et al. [13] could not find support for a unidimensional model based on mothers’ reports of their children aged 10–17 receiving community health supports (i.e., all model statistics used indicated poor fit). With the lack of support for a unitary concept of functioning, Singer et al. [13] returned to EFA to reveal a three-factor model. The first factor was called “school/work”, the second “socializing” and the third “home/family”. However, several large cross-loadings were noted: “getting into trouble” loaded on the first and third factors and “getting along with other kids his/her age” loaded onto the first and second factors. These cross-loadings indicate a lack of full separation into three distinct concepts and raise questions about the structural validity of the scale as a multi-dimensional versus unitary concept that need to be further explored.

Another aspect of measurement that may impact the structural validity of the CIS is how items marked as “not applicable” are treated. For each item in the CIS, the responder can indicate that it does not apply to them. In at least one study undertaken to validate the CIS structure, the analytic sample included only those who provided a response to all items of the scale [11]. In our past research, we found that the non-applicable response option was used by up to 13% of the sample for some items [12]. Further, we discovered that participants who were not in education, employment or training (“NEET”), and who therefore responded ‘not applicable’ to items relating to school and work, had lower functioning based on the mean score of the responded-to CIS items [12]. These findings both raise concern that excluding those with non-applicable responses from the analysis may substantially affect the scale structure that is extracted or confirmed.

Compounding the contradictory evidence for the structural validity of the CIS, to our knowledge, no studies have tested measurement invariance between child- and parent-reported CIS versions. Tests of measurement invariance demonstrate whether, and to what extent, the scale structure fits comparably well across groups of youth and parents. Establishing whether factor loadings (i.e. the strength of relationship between the items and latent constructs) and item intercepts (i.e., the origin of the scale of an item) are equivalent across groups is required, at minimum, to meaningfully compare scale scores between youth and parents [17, 18]. Testing measurement invariance is highly relevant in the context of the CIS since assessment of functioning in relation to psychiatric symptoms is context specific [3, 19] and integrating information from multiple informants who observe varying contexts is advantageous [20–22]. While parent–child agreement for mental health symptoms has shown to be only moderate, multi-informant approaches based on observable aspects of mental health concerns, such as aggressive and disruptive behaviour, appear to offer higher validity [22]. Indeed, the assessment of impairment, in addition to psychiatric symptoms, increases mother–child agreement for psychiatric symptoms [23]. Research on the CIS shows correlation between parents and youth on the total score is moderate overall (*r* = 0.34), but the level of agreement depends on the context being assessed [24]. For example, using Singer’s three factor model, higher correlation was found for home/family (*r* = 0.42), the domain for which youth and parents would be more equally knowledgeable, and lower agreement for socializing (*r* = 0.29), a behaviour that may be less observed by parents [24]. These findings suggest that agreement varies according to context, but this presupposes that the CIS is best represented by a multi-dimensional scale. There is a need, therefore, not only to confirm the dimensionality of the CIS but also determine if it holds across youth and parent reports. Evidence on whether and the extent to which the CIS scale structure is invariant across youth and parents will help inform interpretation of multi-informant data.

The tension between evidence of a unidimensional structure based on EFA findings and lack of support of such a model using CFA creates an impasse in establishing structural validity for the CIS. One path forward, therefore, might be to consider a model that can bring together the two separate approaches to better understand why the EFA and CFA findings do not align. Due to its integration of the best features of EFA with those of CFA Exploratory Structural Equation Modelling (ESEM) is increasingly being used to reveal more complex scale structures [15, 25]. CFA oversimplifies models by constraining all cross-loadings to zero, meaning that each item can only load onto one latent construct, which can make it impossible to confirm scale structures that have been well defined using EFA, since EFA allows for each item to load onto each factor [15]. Misspecification of zero loadings can lead to contorted factors with over-estimated factor correlations, which can misrepresent structural relations [26]. Simulation studies have shown that even cross-loadings as small as 0.1 should be modelled to prevent parameter estimates from becoming inflated and biased [27]. While CFA may be too restrictive, EFA cannot be used to test measurement invariance across groups. Given the contradictory evidence on the structure of the CIS derived from separate attempts to model it using EFA and CFA, ESEM, which unifies the approaches to confirm dimensionality and test measurement invariance, may result in a structure that best represents the underlying data and can be compared across groups. ESEM framework is well suited to explore the inconsistent findings regarding the structure of the CIS and provide a mechanism for dealing the cross-loadings that have been previously report using EFA while allowing for exploration of measurement invariance.

The primary objectives of this study were to apply an ESEM framework to: 1) to better understand the structure of both child- and parent-reported versions of the CIS in a sample of youth 14–17 accessing mental health services and their caregivers without excluding those with non-applicable responses and 2) determine whether measurement invariance across the child- and parent-reported versions can be established.

## Methods

This study comprises a secondary analysis of baseline data collected from the YouthCan IMPACT pragmatic randomized controlled trial [28] (Registration: NCT02836080, Registration Date July 18, 2016). This study was first reviewed by the Centre for Addiction and Mental Health (CAMH) Research Ethics Board (REB), the primary study site, followed by REB approval at the hospital sites and organizational approval at the community sites (see Declarations).

### Sample & setting

The sample included youth and caregiver dyads recruited from one of five outpatient hospital sites in Toronto, Canada between September 2016 and March 2020; while drawn from a similar population, the current sample does not overlap with that used in our previous study [12]. Eligibility criteria included: at least 14 but less than 18 years of age at intake, referral for out-patient mental health and/or addiction services and eligibility for those services. Exclusions criteria included referral to certain specialty services (e.g., forensic services); autism without mental health or addiction problems; primary diagnosis of an eating disorder; active psychosis or imminent risk of self-harm requiring immediate intervention; inability to read and write in English; and inability to consent to the study. Requirements for the caregiver were that they were a regular care provider and familiar with the participating youth’s everyday functioning. Details of the Randomized Control Trial can be found in the protocol [28].

At participating hospitals, clinical administrative staff connected youth seeking out-patient psychiatric services (or their caregiver) with a research staff member, who described the study and screened for interest and eligibility. Interested and eligible participants met with a research staff for an intake meeting to confirm eligibility, obtain informed consent, and provide self-report baseline assessments. A second assessment occurred after randomization, to complete a diagnostic interview. A total of 778 participants were screened by a research staff member, with 247 youth and 189 accompanying caregivers, consented and randomized.

### Measures

The primary outcome measure was the 13-item CIS, with versions for self-report by the youth and proxy report by another informant, a parent or caregiver; we herein refer to the latter group as “caregivers” to ensure inclusivity of the role. Response options range from no problem at all (0) to a very bad problem (4). Respondents can indicate for any given item that it does not apply to them. Despite questions surrounding structural validity, which are the focus on this study, the scale has been shown to have moderate to good convergence validity, internal consistency and test–retest reliability in samples of youth spanning the ages of 9–24 [7, 10, 12, 29, 30] and good to excellent reliability and convergence validity among caregivers [7, 10].

Demographic characteristics of the youth and caregivers were collected in the baseline survey. For youth demographics included age, sex (male, female and not male or female), race (Asian, Black, Latinx, white, multiple, another race not listed), nativity (born in Canada or not), living situation (with or without caregivers), whether mother is the identified caregiver (yes, no) and psychiatric diagnosis using the Diagnostic Interview for Affective and Anxiety Spectrum Disorders – Child Version (DIAS-C) [31]. For caregivers, the same variables were collected in addition to education level (high school or less, trade school or college and university degree), household income and marital status (married/common-law or not).

### Data analysis

The sample characteristics of youth and caregivers were summarized using descriptive statistics. CFA was first undertaken in youth and caregiver samples separately to determine if the original unidimensional structure identified by Bird et al. [7] could be confirmed and, if not, the 3-factor model identified by Singer et al. [13] was tested. The discovery of a less-than-optimally fit model based on CFA provides the empirical justification needed for pursing ESEM and is the recommended first step [32]. ESEM was then undertaken using the better fitting CFA model. Modification indices were used to determine if correlated pairs of residual variances were required for adequate fit. Since the literature provides no guidance on which error terms may need to be correlated, the decision to include any was made cautiously by examining the content of each item [25]. CIS has five response categories, which falls right in the middle with respect to guidance as to whether categories can be treated as continuous [33]. Since more accurate estimation may be achieved using an ordinal specification, especially for items with fewer categories than five and that estimation approaches only converge to produce similar findings at around six to seven categories [33], we opted to use an ordinal specification.

After selecting the best fitting model, invariance was tested across groups using a bottom-up approach that compared nested models assessing configural (factor structure), metric (factor loadings) and scalar (item intercepts/thresholds) invariance. Tests of partial invariance were undertaken when evidence of full invariance could not be found, following recommendations specific to ESEM [17]. We followed the guidance that the majority of items must have both intercept/threshold and loading invariance to make meaningful comparisons across groups [34].

A range of standard fit indices were used to determine model fit. Both excellent and adequate fit thresholds were applied as follows: Root Mean Square of Approximation (RMSEA, < 0.06 and < 0.08), Comparative Fix Index (CFI, > . 95 and > 0.90) and Tucker-Lewis Index [35]. The Weighted Root Mean Square Residual (WRMR, < 1) were also used. The model χ^2^statistic is also reported for completeness but was not used to judge model fit due to its known sensitivity to sample size. Configural invariance, which imposes no equality constraints on parameters, was tested by evaluating the fit of the overall model. Comparison between two nested models was undertaken by assessing changes (Δ) in fit indices. Lack of invariance was determined if CFI and TLI decreases were at least 0.1 or higher or RMSEA increases were at least 0.015 or higher [36]. Following recommendations, more emphasis was placed on TLI and RMSEA comparisons than on CFI, which always produces better fit for more complex models [32].

Analyses were undertaken using Mplus (v7) [14]. As recommended for the modeling of ordinal data, a robust weighted least squares estimator was used [14] with the Delta parameterization [34]. This estimator allows all available data to be used through pairwise deletion so that none of the participants were excluded from the analysis, even if non-applicable responses were selected. While non-applicable responses are not truly missing data, they cannot contribute to the solution in the same way that regularly scored items can (i.e., ordered vs. nominal responses). Using pairwise deletion, if a participant selected a non-applicable response, they would not contribute information to the modeling of data for that particular item but would provide information for all other items. This seemed like a reasonable approach, although it also means that the solution does not integrate the use of not applicable responses. We did not set a minimum threshold for the number of non-applicable responses required to produce a total score. Assessment of missing data revealed almost no incomplete data on the CIS for youth or caregivers, but for almost every item, the non-applicable response option was used. About a quarter of youth (22.3%) and caregivers (25.4%) used the non-applicable response at least once. The items most often responded to with non-applicable were: “getting along with father/father figure” (youth: 8.9%; caregivers: 6.4%) and “getting along with your sister(s) and/or brother(s)” (youth: 8.9%; caregivers: 11.7%).

## Results

### Sample description

In total, we included 189 youth-caregivers dyads. *Youth.* The mean age of youth was 15.7 years (sd = 1.1). Three out of five identified as female; a similar proportion identified as white. Almost nine out of 10 were Canadian born. Most lived with parents and the caregiver was the mother for four out of five participants. The DIAS-C interview revealed the most common diagnosis among participants were mood disorder (64.7%), anxiety disorders (63.4%), attention deficit hyperactivity disorder (30.4%), and obsessive–compulsive disorder (14.3%). *Caregivers.* The mean age of the caregivers was 48.2 (sd = 7.4). Almost nine out of 10 identified as female. Half had a university degree, and most were Canadian born. Three-quarters identified as white; just over one in 10 were Asian; a similar proportion were Latinx. Almost half had a household income of ≥ $120,000 and most were married or common-law. See Tables 1 and 2 for details of the sample.

**Table 1:** Description of sample of youth

	n (%)
Age	
14 years	42 (22.3)
15 years	58 (30.9)
16 years	47 (25.0)
17 years	41 (21.8)
Sex	
Male	68 (36.0)
Female	116 (61.4)
Not male or female	5 (2.7)
Born in Canada	
No	24 (12.7)
Yes	165 (87.3)
Race	
Asian	19 (10.4)
Black	8 (4.4)
Latin American	12 (6.6)
Mixed	16 (8.7)
White	115 (62.8)
Other	13 (7.1)
Living with parents	
No	7 (3.7)
Yes	182 (96.3)
Mother is caregiver	
No	29 (15.3)
Yes	159 (84.6)

**Table 2:** Description of sample of caregivers

Caregivers (*n* = 189)	
	n(%)
Sex	
Male	19 (10.1)
Female	169 (89.4)
Not male or female	1 (0.5)
Relationship to youth	
Mother	164 (86.8)
Father	17 (9.0)
Other	8 (4.2)
Highest level of education	
High school degree or less	36 (19.1)
Trade school/college	49 (25.9)
University degree	93 (49.2)
Other	11 (5.8)
Born in Canada	
No	70 (37.0)
Yes	119 (63.0)
Race	
Asian	20 (10.6)
Black	4 (2.1)
Latin American	18 (10.6)
Mixed	5 (2.7)
White	127 (74.7)
Other	14 (7.5)
Household income	
< $30,000	29 (15.8)
$30,000–59,000	29 (15.8)
$60,000–89,999	13 (7.1)
$90,000–119,999	26 (14.1)
$120,000—149,000	25 (13.6)
$150,000 or over	62 (33.7)
Marital status	
Married/common-law	130 (75.6)
Not married or common-law	42 (24.4)

### Confirmatory factor analysis

The unidimensional model had inadequate fit for both youth and caregivers (see Table 3). Singer’s three-factor model [13] was better, but fit was less than adequate on most fit statistics in both groups. On balance, Singer’s three-factor model of “home/family”, “school/work” and “socializing”, was the best fitting and was selected for further tested using ESEM to obtain better fit.

**Table 3:** Comparison of CFA and ESEM models for Separate Groups of Youth and Caregivers

		Youth					Caregivers				
		RMSEA (90% CI)	CFI	TLI	WRSR	**χ**^**2**^** (*df*)**	RMSEA	CFI	TLI	WRSR	**χ**^**2**^** (*df*)**
Model	*CFA Models*										
1a	unidimensional	0.158 (.143-.174)	0.7	0.639	1.66	373.57 (65)*	0.166 (.151-.182)	0.834	0.801	1.648	403.83 (65)*
1b	3 factors	.089 (.072-.107)	0.909	0.886	1.017	155.17 (62)*	0.127 (.110-.143)	0.908	0.885	1.177	249.55 (62)*
	*ESEM Models*										
1a	3 factors	.059 (.033-.083)	0.973	0.95	0.532	69.71 (42)*	0.088 (.067-.110)	0.97	0.944	0.574	103.53 (42)*
1b	3 factors with correlated residuals (7&10)	0.049 (.016-.075)	0.982	0.965	0.485	59.82 (41)*	0.079 (.056-.101)	0.977	0.956	0.515	88.8 (41)*

^*^chi square is statistically significant

### ESEM in each group separately

The initial model fit for the three-factor structure obtained using ESEM was better among both youth and caregivers than that found using CFA. For most fit statistics, fit was shown to be at least adequate for both groups (see Table 3). Modification indices indicated a path between the error terms of items 7 and 10 for both youth and caregivers, 5 and 12 (youth), 7 and 12 (youth) and 10 and 12 (caregivers) would improve fit. After reviewing the content of the item pairs, correlated errors were deemed justified for items 7 and 10 because both items measured “getting along with others”; the other items were left uncorrelated because the content of the items were unrelated or the need for correlated errors was only demonstrated in one sample.

Factor loadings are presented for the most optimal model for youth and caregivers separately in Table 4. The pattern of loadings was well defined (0.35—0.92) and consistent with the 3-factor model identified by Singer et al. [13] of “home/family”, “school/work” and “socializing” factors, with the latter factor mapping onto identical items in both studies. Despite well-defined factors, cross-loadings were present, some quite large (max = 0.41). However, when considering the underlying construct, most cross-loadings were reasonable. For example, the item “getting into trouble” cross-loaded on “school/work” and “home/family” among caregivers.

**Table 4:** Standardized Factor Loadings from a 3 factor ESEM Model in samples of youth and caregivers

	Home/family		School/work		Socializing	
	Youth	Caregivers	Youth	Caregivers	Youth	Caregivers
1. Getting into trouble	0.206	0.749	0.693	0.307	-0.014	-0.23
2. Getting along with mother	0.988	0.963	-0.118	-0.15	-0.003	-0.056
3. Getting along with father	0.689	0.812	0.022	-0.184	-0.142	-0.001
4. Feeling unhappy or sad	0.079	0.363	-0.012	-0.072	0.727	0.543
5. Behavior at school/job	0.002	0.34	0.743	0.579	0.248	-0.003
6. Having fun	-0.007	0.15	-0.166	0.007	0.87	0.766
7. Getting along with non-parental adults	-0.054	0.65	0.435	0.125	0.222	0.068
8. Feeling nervous or afraid	-0.054	-0.044	0.145	0.14	0.585	0.638
9. Getting along with siblings	0.5	0.605	-0.062	-0.002	0.056	0.086
10. Getting along with other children	0.047	0.236	0.157	0.225	0.475	0.458
11. Getting involved in activities and hobbies	0.015	-0.002	0.184	0.409	0.54	0.564
12. Doing school/job work	0.089	0.003	0.353	0.82	0.261	0.118
13. Behaviour at home	0.581	0.861	0.099	0.018	0.099	0.049

### Measurement invariance

The most optimal model from the previous ESEM step, i.e., the three-factor model, was selected to test measurement invariance. The configural model fit was good, suggesting an equivalent factor structure across groups (see Table 5). Next, the equality of factor loadings was tested; loading invariance was found based on the ΔTLI/CFI ≤ 0.010 and ΔRMSEA ≤ 0.015. The equality of item thresholds were tested next by comparing the latter model to a one that applied constraints to the threshold, which can be thought of the boundaries of each category representing the response options for the CIS items on a continuous scale.

**Table 5:** Measurement Invariance Models in a sample of youth and caregivers

	Model	χ^2^ (*df*)	CFI	TLI	RMSEA (90% CI)	Model comp	Δχ^2^ (Δ*df*)	ΔCFI	ΔTLI	ΔRMSEA	Decision
M1: Configural Invariance	1	149.78 (83)*	0.98	0.962	0.061	–-	–-	–-		–-	–-
M2: Metric Invariance	2	209.38 (113)*	0.971	0.96	0.063	1	59.6 (30)	-0.009	-0.002	-0.002	Accept
M3: Scalar invariance	3	343.76 (162)*	0.945	0.947	0.072	2	66.54 (49)	-0.026	-0.013	-0.009	Reject
M3p: Partial scalar invariance	4	294.31 (158)*	0.959	0.959	0.063	2	17.09 (6)	-0.012	-0.001	0	Reject

^*^chi square is statistically significant

However, evidence supporting threshold invariance was weak, with both ΔTLI/CFI > 0.010, suggesting that the category boundaries did not align on a continuous scale in the same way across groups. Modification indices were consulted to determine if partial invariance could be established by relaxing the equality constraint of one or more items’ thresholds. Partial invariance at the threshold level is when some thresholds are allowed to vary across groups. With the relaxation of a single item’s thresholds (item 1), some evidence in favour of partial threshold invariance was found (ΔTLI < 0.010 and ΔRMSEA ≤ 0.015), although ΔCFI was borderline (-0.012). While it was possible to look for more thresholds to freely estimate in both groups, we stopped here. This decision was justified by our desire to make minimal post-hoc modeling decisions that are unlikely to generalize to other samples.

## Discussion

The unidimensional structure originally espoused by the CIS developers [7] and most frequently demonstrated in previous research [11, 12] could not be confirmed in a sample of youth aged 14-to-17 years seeking mental health or substance use services or in a sample of their caregivers. The three-factor model previously defined by Singer et al. [13] using EFA also could not be confirmed among youth or caregivers with CFA, but a three-factor model was supported, in both groups, using ESEM. ESEM allows for cross-loadings to be modelled, which can result in better fit and a more realistic representation of the data for models that do not obtain adequate fit using CFA [15].

Large cross-loadings are present in the current solution, as they were in Singer et al. [13] three-factor EFA model and call into question how best to interpret the scale.. Consistent with Singer et al. [13], the item “getting in trouble” loaded on multiple scales: “home/family” and “school/work”. This is a conceptually relevant cross-loading: “getting into trouble” is not limited to one domain and may in fact reflect a more general aspect of impaired functioning [25]. For example, deficits in impulse control may have broad sequelae across multiple contexts and therefore represent a fundamental aspect of functioning. However, other cross-loadings were less meaningful. For example, “behaviour at school/job” cross-loaded between home/family and school/work. On one hand, the presence of the cross-loadings in the CIS may suggest that functioning is not a simple concept to measure, pointing to the need for a complex measurement model that accounts for the general aspects of functioning through broad impairments (e.g. executive functioning, impulse control) in addition to context-specific features (e.g., socializing). Such a model is not unlike transdiagnostic models of depression and anxiety that contain common cognitive vulnerabilities in addition to disorder-specific features [37]. On the other hand, it is possible that the items in the CIS simply do not promote complete separation of concepts. In which case, there could be a need revise certain items to be better indicators of underlying constructs [25]. Either way, our finding that the unidimensional model provides poor fit suggests the routine use of the global summary score may need to be re-evaluated. It will be important to test the bifactor model to determine if “essential unidimensionality” (i.e. enough unity in the concept) can be established to justify use of a global score [38]. Until this work can be undertaken, approaches that can accommodate use of latent factors, such as Structural Equation Modelling, remain the most valid and reliable way to incorporate functioning as measured by the CIS into analyses.

This is the first study to test the measurement invariance of the CIS across multiple groups. Measurement invariance between youth and parents was observed for the three-factor model at the configural level and the level of the factor loadings. However, measurement invariance was not observed at the level of the item thresholds. We attempted to determine if partial measurement invariance could be found by relaxing the equality constraints across groups. For example, the item “getting into trouble” was found to be non-invariant and relaxing the equality constraints made the model fit better. Youth had a lower threshold (I.e., the boundaries for the response categories) than caregivers, which can be interpreted as youth being more likely to report getting into trouble. This is consistent with previous research demonstrating marginally higher ratings on the item getting into trouble among youth compared to parents [24]. Likely this reflects differences in vantage points: caregivers may not know the full extent of the trouble youth have experienced. Indeed, research has demonstrated that reported nonaggressive symptoms (i.e. lying, school truancy) of conduct disorder are moderated by informant [39]. On one hand this highlights the importance of collecting reports of these behaviours from multi-informants [40]. On the other it raises questions about how scores can be compared if they are not measuring the same thing. Unfortunately, relaxing the equality constraints of the thresholds for the getting in trouble item alone did not provide strong enough evidence of invariance. And while we could have kept trying to find more items thresholds to freely estimate in both groups, we stopped at this point. Morin et al. [17] warn that the post-hoc development of partially invariant models should be interpreted with caution as they can reflect idiosyncrasies of the characteristics of the particular sample. Future research is needed to replicate our findings and determine if a model based on partial scalar invariance can be developed and generalized across samples.

This research has many strengths, including a moderately large sample of youth-caregiver dyads who both completed the CIS. The sample also represents diverse clinical presentation of youth with a psychiatric disorder across a geographically large urban centre. However, it is not without some limitations. First, parents were primarily mothers, and it is unknown how well the findings would be replicated in samples of fathers or other types of familial or non-familial caregivers. Future research should formally address this question, as differential reporting according to gender of the parent and child has been observed for some behavioural problems [41].

Second, our sample represent a narrow age group of 14-to-17 years. Singer et al. [13] similarly used a sample with a maximum age of 17. However, our previous research, where we found evidence of a unidimensional model using EFA, was based on an older sample, up to age 24 (mean age 19.2). We also found that items about relationships with mother and father figures had low loading values, suggesting that they did not correlate well with the other items [12]. Although this needs to be tested formally, it is possible that the CIS has different measurement properties in samples of younger children/youth for whom a parent or caregiver is still centrally involved in their life. In future research, it will be important to apply a development lens to determine if there are upper age bounds for which the multidimensional scale structure remains valid.

Third, the CIS enables individuals to provide a response of not appliable to any item. This is because contexts are not experienced identically. For example, not everyone has a sibling and not all youth may be working or in school. Consequently, the number and types of items used to measure functioning may vary across different groups of individuals. We dealt with this issue by treating not applicable responses as missing and applying pairwise deletion to use all available data to model the data. However, not applicable responses are not truly missing data and may be better dealt with by treating it as a nominal response embedded within the ordered response options [42]. While frameworks for analysis of scales featuring not appliable options exist (e.g. [42]), it is not clear how they best can be integrated into the ESEM model we used. Testing measurement invariance across groups based on different patterns of use of the non–applicable response option could be undertaken, although this would lead to comparison of many different models. It does, however, beg the question as to if the concept of functioning as measured by the CIS is the same regardless of the particularities of the contexts within which it is assessed.

To conclude, through application of the ESEM framework to better understand the structure of the CIS, we found a multi-dimensional model best fit the data that allowed for both distinction of school/work, home/family and socializing domains and generality as evidenced by some large item cross-loadings (e.g. getting into trouble). This finding calls into question whether and how the global scale can best be used in clinical and research settings. Further, the demonstrated lack of evidence of scalar invariance reinforces the need to interpret multi-informant data carefully. For example, concepts like getting into trouble may mean slightly different things to youth and their caregivers. An important next step will be to test the CIS for essential unidimensionality.

## Data Availability

The data that support the findings of this study are not openly available due to reasons of sensitivity and are available from the corresponding author upon reasonable request and upon execution of a data sharing agreement. Data are located in controlled access data storage at the Centre for Addiction and Mental Health.

## Abbreviations

CIS: Columbia impairment scale
CFA: Confirmatory factor analysis
EFA: Exploratory factor analysis
ESEM: Exploratory structural equation modelling
MLE: Maximum likelihood estimator
NEET: Not in education, employment, or training
